# Extensive structural change of the envelope protein of dengue virus induced by a tuned ionic strength: conformational and energetic analyses

**DOI:** 10.1007/s10822-012-9616-4

**Published:** 2012-11-18

**Authors:** Léo Degrève, Carlos A. Fuzo, Antonio Caliri

**Affiliations:** 1 Departamento de Química, Faculdade de Filosofia, Ciências e Letras de Ribeirão Preto, Universidade de São Paulo, Av. Bandeirantes, 3900, Ribeirão Preto, SP 14040-901 Brazil; 2 Departamento de Física e Química, Faculdade de Ciências Farmacêuticas de Ribeirão Preto, Universidade de São Paulo, Av do Café S/N, Monte Alegre, Ribeirão Preto, SP 14040-903 Brazil

**Keywords:** Flavivirus, Dengue, Molecular simulation, Ionic strength, Protonated histidine, Inter-domains angle, Hydrogen bonds

## Abstract

The Dengue has become a global public health threat, with over 100 million infections annually; to date there is no specific vaccine or any antiviral drug. The structures of the envelope (E) proteins of the four known serotype of the dengue virus (DENV) are already known, but there are insufficient molecular details of their structural behavior in solution in the distinct environmental conditions in which the DENVs are submitted, from the digestive tract of the mosquito up to its replication inside the host cell. Such detailed knowledge becomes important because of the multifunctional character of the E protein: it mediates the early events in cell entry, via receptor endocytosis and, as a class II protein, participates determinately in the process of membrane fusion. The proposed infection mechanism asserts that once in the endosome, at low pH, the E homodimers dissociate and insert into the endosomal lipid membrane, after an extensive conformational change, mainly on the relative arrangement of its three domains. In this work we employ all-atom explicit solvent Molecular Dynamics simulations to specify the thermodynamic conditions in that the E proteins are induced to experience extensive structural changes, such as during the process of reducing pH. We study the structural behavior of the E protein monomer at acid pH solution of distinct ionic strength. Extensive simulations are carried out with all the histidine residues in its full protonated form at four distinct ionic strengths. The results are analyzed in detail from structural and energetic perspectives, and the virtual protein movements are described by means of the principal component analyses. As the main result, we found that at acid pH and physiological ionic strength, the E protein suffers a major structural change; for lower or higher ionic strengths, the crystal structure is essentially maintained along of all extensive simulations. On the other hand, at basic pH, when all histidine residues are in the unprotonated form, the protein structure is very stable for ionic strengths ranging from 0 to 225 mM. Therefore, our findings support the hypothesis that the histidines constitute the hot points that induce configurational changes of E protein in acid pH, and give extra motivation to the development of new ideas for antivirus compound design.

## Introduction

Dengue is an acute infectious disease occurring in tropical and sub-tropical areas of the world, affecting people of all ages. It is becoming a major public health issue worldwide: Nowadays, the disease is endemic in over 100 countries with 2.5 billion people living in risk areas; roughly, 100 million infections occur annually with about 900,000 cases in the Americas. Approximately 3 % of these cases develop the most severe forms of the disease, occurring after a secondary infection by a different strain of dengue virus (DENV), which places people at risk of developing dengue hemorrhagic fever and dengue shock syndrome [1]. These are severe dengue-related complications connected with the phenomenon called “antibody-dependent enhancement of infection”, on account of the existence of four DENV serotypes, labeled as DENV-1, DENV-2, DENV-3 and DENV-4 [2]. The virus is mainly transmitted by the bite of an infected *Aedes* mosquito and, to date, there is no licensed vaccine available for it.

DENV is a single positive-stranded RNA (10.7 kb) virus member of the *Flavivirus* genus of the *Flaviviridae* family (arbovirus type B) [3]. The DENV consists of an external glycoprotein shell and an internal lipid bilayer (host-derived) that encloses an RNA–protein core comprising the capsid (C) proteins, which in its turn contains the genetic material inside [4]. The outer glycoprotein shell is composed of 180 copies of an envelope (E) glycoprotein, each *anchored in the lipid* bilayer through a transmembrane (M) protein. In mature DENV they are found as 90 homodimers that lie flat against the viral surface, an icosahedrally symmetric ectodomain about 50 nm in diameter [5].

The E proteins play important roles in all stages of the infection course, from the first contact with the host cell up to the delivering of the genetic material within the cell. The complex course of the virus replication is initiated in the endosome, where the E proteins start the procedure that triggers the membrane fusion process [6, 7]. Considering the crystallographic structures of the E protein, both pre-fusion (dimers) and post-membrane fusion (trimers), it has been suggested that once in an environment subjected to a progressive reduction of pH (reaching pH about 6.5), the E protein dimer dissociates [8] and undertakes a large conformational rearrangement that allows for the formation of trimers [9]. This process roughens the surface of the virus facilitating the progression of membrane fusion. Therefore, at some point along this process, at least a fraction of E proteins assumes a monomer form in an acid medium and ionic strength of around 150 mM.

Being fully exposed to the medium, the E proteins have been considered as the main antigenic target on the surface of the DENV: indeed, although the epitopes responsible for human neutralizing antibodies have not yet been identified, it has been reported that many monoclonal antibodies (MAb) that bind to E protein strongly neutralize DENV [10]. This finding in itself suggests that important dividends can be gained by a detailed conformational description of the E protein at the molecular level. However, there are other reasons that support this proposition, such as the existence of four DENV serotypes, and the fact that, despite the close similarities between DENV and yellow fever virus, for which there is a commercially vaccine that has been used for over 50 years, a dengue vaccine is still unavailable, neither specific antiviral medicines [11–19].

The E protein structure, rich in β-sheets, is composed of three domains, labeled as domain I (DI), DII and DIII [7, 10, 15]. DII is an elongated structure bearing a hydrophobic sequence at its tip (a loop that in DENV2 involves residues Asp98–Gly109), which is conserved among all flaviviruses. DI forms a central, stranded β-barrel, which contains a single N-linked glycosylation site. DIII is characterized by its immunoglobulin-like fold; through its C terminal, the E protein is anchored to the virus membrane by the transmembrane (M) protein. Particularly, DIII has received much attention: it is somewhat projected above the viral surface and is the only domain that can exist independently of the rest of the protein chain. Additionally, mouse MAbs that bind DIII, either serotype-specific and highly neutralizing [10, 20], or cross-reactive and non-neutralizing MAbs [21] can modify the arrangement of E proteins, suggesting that they are relatively free to do translation and rotational movements on the viral surface. It is also well established experimentally that the E protein undergoes extensive conformational change during the infection process [7]. Therefore, overall flexibility seems to be a functional characteristic for assembling and infection of DENV [22].

Crystallographic, NMR and other complementary methods have provided essential structural data of the E protein of DENV. We have basically a picture of the E protein ectodomain before (dimers) and after fusion (trimers). However, to accomplish its diverse functions, the E protein must exhibit some conformational and dynamical peculiarities at single molecular level, which cannot be investigated with the employment of such traditional experimental techniques. Therefore, in this work, we employ Molecular Dynamics (MD) simulations to characterize structural and dynamically the E protein under distinct intensive thermodynamic parameters. Our main goal is to identify the thermodynamic conditions in which the E proteins are induced to experience extensive structural changes, such as during the process of reducing pH [23–26].

The basic general strategy of the molecular simulation to the DENV problem, as adopted here, is to focus on a specific protein that acts outside the cell, or on a protein that plays a key role in the process of membrane fusion, trying to find potential sites whose corresponding activity can be inhibited by ligands, which have yet to be identified. To implement such purposes, knowledge regarding the three-dimensional structure of the protein at the atomic level is imperative, and the E protein ectodomain is the only one that meets all such requirements.

## Methods

The systems were settled by inserting the E protein monomer of the DENV2 (pdb id 1oke) in the center of a parallelepiped box of sides 8.0, 9.0 and 16.0 nm. The E protein structure was determined by X-ray diffraction of crystals produced at basic pH in a medium of ionic strength greater than 1 M (one molar); pdb id: 1oke and 1oan [25, 26]. It is dimeric in solution as well on the viral membrane surface [22], but at low pH it is destabilized producing monomers, such as have been demonstrate experimentally [25, 26] and by MD simulation [27]. Therefore, we adopt the atomic coordinates of the chain-A of the structure of E protein, which will be called Xtal hereafter, as the configuration for starting our MD simulations. The RMSD between 1oke and 1oan is 0.11 nm, so we can accept that these structures are equivalent for our purpose. The monomer was included in the simulation box, which was supplemented with water (about 36,000 molecules) represented by the model SPC/E [28], and appropriate amount of ions to neutralize the system. Simulations were performed using version 4.5.1 of the gromacs package [29–32] at 300 K and 1 atm using the Berendsen algorithms to control temperature and pressure [33] with correlation times of 0.5 ps for the pressure and 0.01 ps for the temperature controls. The specific physiological conditions of extra cellular medium are: acidic pH, ionic strength of about 150 mM and cations that are predominantly Na^+^ [33, 34]. The histidine residues are firstly considered in their full protonated form, at the ND1 and NE2 positions, and four media were considered, namely, ionic strength zero, 75, 150 and 225 mM, which were adjusted by the inclusion of different numbers of Na^+^ and Cl^−^ ions. Cut-offs on the electrostatic interactions were applied at 1.2 nm, and the smooth particle mesh Ewald method [35] was applied to complete the energy calculations. The atom interactions were described by the GROMOS96 43a1 force field. The structure of water molecules was preserved through the SETTLE algorithm [36], and the bonds with hydrogen atoms maintained with the aid of the LINCS algorithm [37]. The simulation times after the startup phase were 330, 250, 400 and 350 ns for the systems with ionic strength zero, 75, 150 and 225 mM, respectively. The average internal energies for these systems at completing the simulation were −348, −359, −366 and −377 × 10^6^ kcal/mol, respectively, with deviations of approximately 0.05 % when using a 2 fs integration step—the energy unit employed, kcal/mol, means that the whole system energy (protein and solvent molecules) obtained in the simulated system was multiplied by the Avogadro number.

**Fig. 1:**
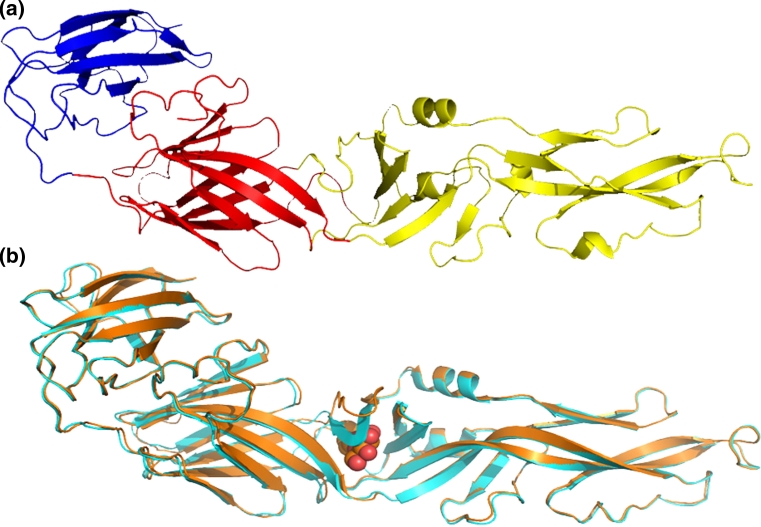
**a** Experimental structure of the monomer of E protein (pdb id: 1oan). All domains DI (*red*), DII (*yellow*), and DIII (*blue*) contain several beta strands conferring strong structural conformation to each of them. **b** Superposition of the experimental structures 1oan (*cyan*) and 1oke shows that the binding of β-OG molecule (*red spheres*)—at the boundary of DI and DII—causes a minor locally rearrangement of the β-hairpin (residues 268–280)

The monomer of the E protein is an elongated conical molecule, about 13 nm high and a base diameter of 4 nm. It consists of 394 residues (including 11 histidine residues, five of which are absolutely conserved among all four serotypes) distributed in three domains, DI, DII and DIII. Six disulfide bridges are present between pairs of cysteine residues [25, 26]. The secondary structures of the E protein consist mainly of β sheets; details are given in Table 1, as well as the respective mean energies (kcal/mol) of the hydrogen bonds (HB) that form the corresponding structures. The mean energy per residue highlights the importance of β sheets in maintaining the protein structure. Two experimental structures of the E protein, namely pdb id 1oke and 1oan, are shown in Fig. 1.

**Table 1:** Secondary structures of the E protein monomer

Type	Strands		Energy (kcal mol^−1^ residue^−1^)	Type	Strands		Energy (kcal mol^−1^ residue^−1^)
	A	B			A	B	
β1+	9–13	30–34	−11	β7−	182–186	284–288	−12
β2−	20–25	283–288	−14	β13−	196–200	126–128	−16
β3−	31–33	41–43	−16	βl4−	198–200	205–209	−17
β4−	41–50	135–143	−14	βl5−	205–207	268–271	−8
β8−	54–72	113–129	−12	β16–	238–241	249–252	−12
β9−	57–59	220–222	−13	αHl	256–260		−3
β10−	69–72	113–116	−15	βl7−	306–314	320–326	−13
β11−	90–99	109–118	−15	β18−	320–324	365–369	−13
β12−	126–128	198–200	−15	β19−	337–339	378–380	−12
β05−	138–141	160–163	−13	β20−	365–369	320–324	−11
β06−	171–175	179–183	−13	β21−	374–380	387–393	−13

The first column labels the type of secondary structure: parallel β-sheet (β+); antiparallel β-sheet (β−) and α helix (αH). Columns **A** and **B** identifies the residue sequences that form the secondary structure; the last column gives the mean HB energy (kcal/mol) per residue in each particular structure. The short helices, Glu84–Glu85 and Gln211–Leu214, which can be visually detected in Fig. 1 do not obey the criteria of Reference [40]

Images were created using the Pymol free version (available at: http://www.pymolwiki.org/index.php/Main_Page), Swiss-PdbViewer (available at http://spdbv.vital-it.ch/) and VMD (available at: http://www.ks.uiuc.edu/Research/vmd/).

## Results

### Structural analysis

**Fig. 2:**
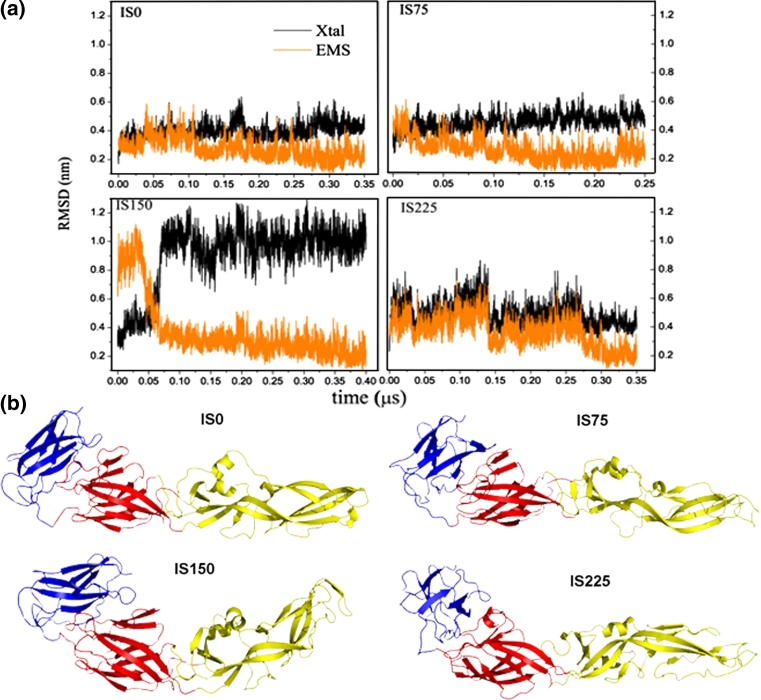
**a** RMSD evolution is shown for all four different systems, at pH = 7. *Black* and *orange curves* correspond to RMSD relative to the experimental (Xtal) and EMS structure, respectively. At about 0.06 μs, the system IS150 consolidates an extensive conformational change, which is maintained up to the end of the simulation (0.4 μs). For IS0 and IS75 systems, the structures persist quite stretched, while system IS225 is the one that presents the largest oscillations. Note that the lowest RMSD fluctuation occurs in the IS75 system. **b** EMS configurations of the E protein for the four systems with different ionic strength. Note that the EMS, for the IS150 system, is bended in the hinge region between DI and DII, approaching DII towards DI/DIII in relation to the Xtal structure

We employed the Root Mean Square Deviation (RMSD) of Cα atoms in order to examine the configurational evolution of the monomeric E protein in solution at acid pH. We use as reference the experimental structure (Xtal) and the Ensemble Mean-Structure (EMS), which was composed by the last 50 ns of the corresponding MD simulations. The four systems considered, with ionic strength zero, 75, 150 and 225 mM, will henceforth be referred to as IS0, IS75, IS150, and IS225 respectively. Figure 2a, b show, for the four systems, respectively, the MD configurational evolution of the E protein with respect to the Xtal and EMS, and EMS final configurations. RMSD between Xtal and EMSs and between pairs of EMSs, for all four systems, are summarized in Table 2.

**Table 2:** Comparative RMSD-matrix of structures deviations

	Xtal	ISO	ISO75	IS15O	IS225
Xtal	0	0.41	0.56	**0.90**	0.55
ISO	0.41	0	0.50	**0.84**	0.68
IS75	0.56	0.50	0	**1.01**	0.73
IS15O	**0.90**	**0.84**	**1.01**	0	**1.10**
IS225	0.55	0.68	0.73	**1.10**	0

The symmetric Matrix elements M_*p*,*q*_, with *p* = 0 and *q* = 1 up to 4, correspond to the RMSD (nm) between the crystallographic structure (Xtal) and EMS of all four distinct systems, namely IS0, IS75, IS150 and IS225 systems. Matrix elements M_*p,q*_ with *p* and *q* > 0, give the RMSD between all pairs of EMSs of the systems. The smaller RMSD (for *p* ≠ *q*) is that between the Xtal and IS0 configurations, and the lagers RMSD always involve the IS150 system (bold figures)

The lowest RMSD level with respect to the crystallographic structure is found in the IS0 system; it increases until the IS150 and then decreases back in IS225. Two opposing factors determine these profiles: the influence of the initial configuration, taken from the crystallographic coordinates of the extended dimeric E protein, and the ionic strength—that increases from zero up to 225 mM, approaching to the experimental setup employed for obtaining the protein crystals (at pH ~8, when the histidine residues are not protonated, and IS > 1 M). In the IS0 system, the protein conformation deviates from Xtal structure, with the RMSD-Xtal oscillating about 0.4 nm, and peaks reaching 0.6 nm. It converges to conformations, which, to some extent, are different from the EMS: the RMSD-EMS oscillates in phase or in phase opposition with RMSD-Xtal, suggesting the occurrence of cooperative movements of the molecule.

For the IS75 system, the RMSD-Xtal evolution shows that after an initial relaxation (with RMSD-Xtal about 0.5 nm) the structure remains fairly stable. On the other hand, initially the RMSD-EMS oscillates recurrently: between 0.15 and 0.22 μs the structure presents the smallest deviations, while at the end of the simulation it becomes a little wobbly. However, both RMSDs suggest that the relaxed structure is very stable; the recurrent RMSD-EMS oscillations occur because unstable sectors of the protein (loops, for example) are represented by average conformations in EMS.

Under the IS225 system conditions, a large and *quasi*-periodic RMSD oscillation indicates that the protein structure is relatively free to populate alternative conformations: high ionic concentrations increase the shielding effect of the electrostatic interactions, making the protein structurally less stationary. Along the simulation, three abrupt conformational transitions are observed, bringing the chain gradually closer to the Xtal configuration, which is followed by a slow relaxation to another conformation.

**Fig. 3:**
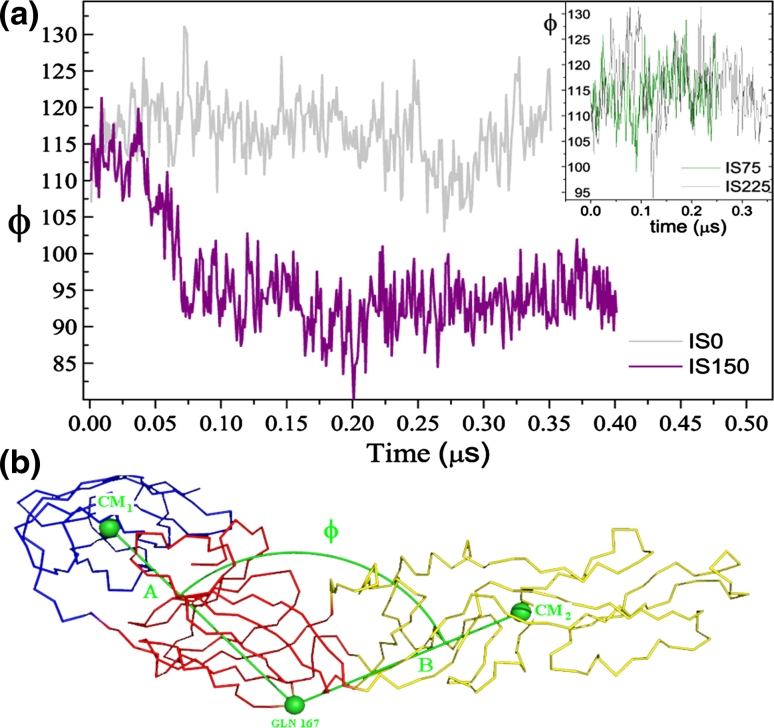
**a** Comparative evolution of angle ϕ (defined in part B) between systems IS0 (*gray line*) and IS150 (*purple line*): low pH and specific ionic strength induce a large conformation change in the E protein. At about 0.06 μs, the system IS150 consolidates an extensive conformational transition, changing the average angle ϕ from about 112 down to about 95°. In the system IS75 and IS225 (*inset* of part **a**) the chain remains stretched, but in the latter (*gray line*) configurational fluctuations are larger. **b** Definition of the ϕ angle: ϕ is the smaller angle formed by the intersection of segment *A* and segment *B*: segment *A* connects the center of mass of DIII (CM_1_) to residue Gln167, and segment *B* connects residue Gln167 to the center of mass of DII (CM_2_). DI and DIII are practically motionless, relatively to one another

**Fig. 4:**
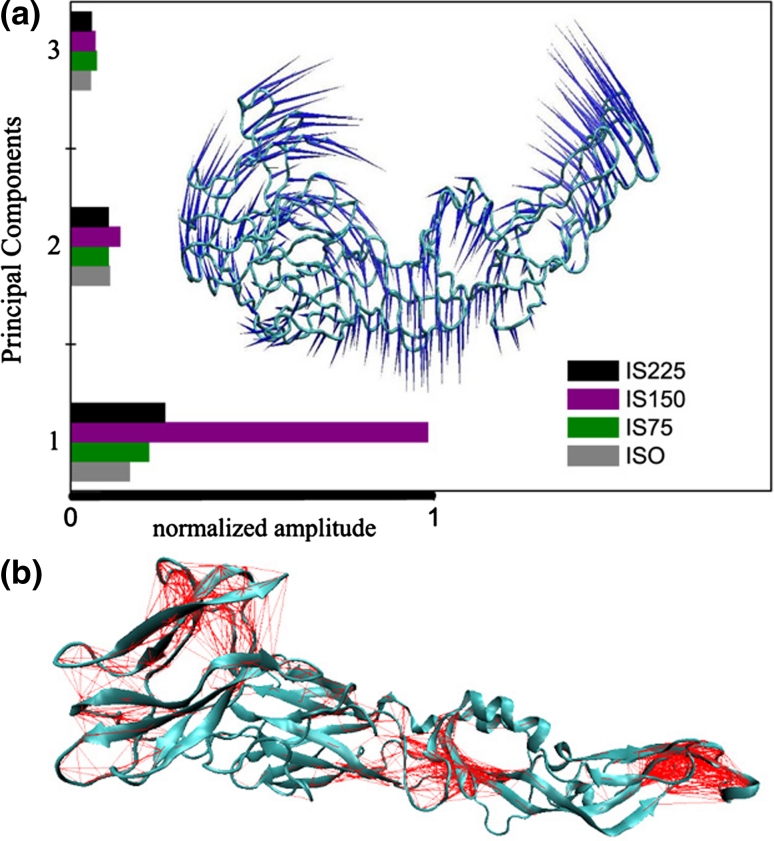
**a** Porcupine plot showing likely modes of large-scale collective motion for system IS150. The orientation and length of the blue cones indicate the direction and the relative amplitude of motion of the C_α_ atoms. The normalized histogram (*colored horizontal bars*) shows the relative size of the three first eigenvalues for all four systems. Note that only for the system IS150 the first eigenvector is much larger than the others, indicating that the E protein motion is dominated by the first eigenvector. **b** Plot of atomic correlation (principal component analysis at a threshold of 90 %). The high correlations between pairs of C_α_ atoms (*red lines*) identify those protein regions that move as rigid bodies. Note that the three rigid regions of E protein determine two other regions, more flexible: the center of DII and almost all DI

Lastly, we observe that the IS150 system demonstrates a peculiar behavior: DI and DIII remain conformationally stable in *relation to* *one* another, but an extensive structural change occurs between them and DII, as indicated by the evolution of its RMSD (Fig. 2a) with respect to the Xtal structure and EMS. Now, Fig. 3a shows details of that conformational change through the temporal evolution of ϕ, defined as the angle between the intersection of segment A, which connects the center of mass of DIII (CM_1_) to residue Gln167, and segment B connecting residue Gln 167 to the center of mass of DII (CM_2_), as depicted in Fig. 3b. The residue 167 was chosen for being fairly stable with respect to DI/DIII and very close to the protein hinge. The average ϕ drops from about 112° to approximately 90° remaining stationary until the end of the simulation, at 0.4 μs. This result suggests that, at certain acid pH and physiological ionic strength about 150 mM, the E protein can attain a new structural conformation (approximately 50 ns after the simulation begins), processed by an explicit move of DII toward DI/DIII; Fig. 2b. The conformational transition displayed by the IS150 system, Fig. 3a, follows from collective motions: Fig. 4a shows the predicted first eigenvector of motion pictured as a porcupine plot of the principal component analysis [38]. The porcupine plot is based on the system IS150 trajectory generated with Gromacs (400 frames separated in intervals of 1 ns) and analysed using DynaTraj [39]. It corresponds to an outline of E protein motion, suggesting what part of the protein moves in concert and in which direction. Figure 4a also shows the (normalized) first three larger eigenvalues for all four systems: note that only for the IS150 there exists a predominant eigenvector much larger than all others, meaning that the motions and relative amplitudes shown in the porcupine plot are representative of the movements of the protein. Figure 4b, in its turn, shows the plot of atomic correlation[38] at a threshold of 90 %. The high correlation between pairs of C_α_ atoms in three non-adjacent structural subunits indicates that these regions can move as rigid bodies. Notably, the fusion peptide, at the distal tip of DII, is one of these stiff regions of the E protein, as well as the DIII, located at the opposite extreme of the E protein. The third compact region is seen in the other extreme of the DII, adjacent to DI. Therefore, under the environmental condition considered here (acid pH and IS150), two regions of E protein turn out to be more flexible: one is located in the middle of DII, and the other region corresponds to the most part of DI; Fig. 4b. The correlation images were generated using the same trajectory (composed of 400 frames) used for Fig. 4a.

**Fig. 5:**
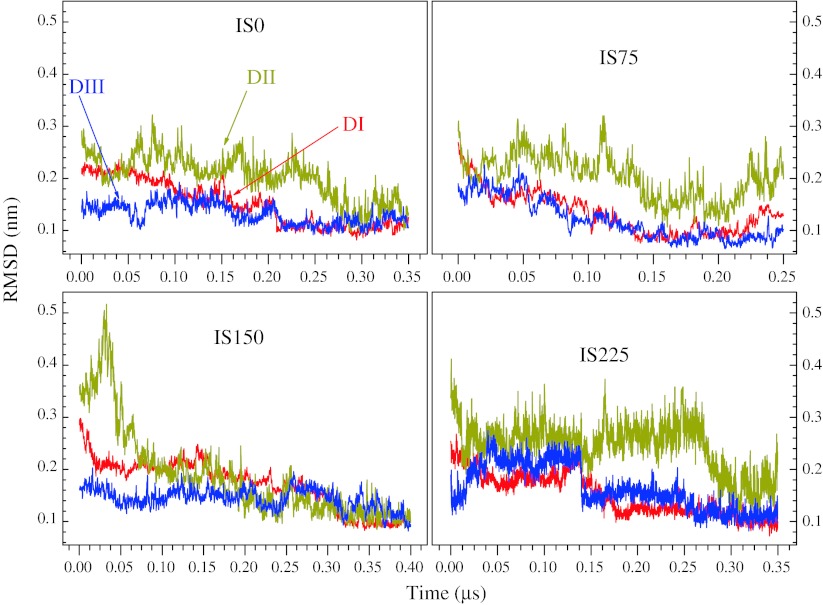
Evolution of the RMSD for all four systems and for the three domains: *red*, *dark yellow* and *blue* represent DI, DII and DIII, respectively. In all cases the RMSD converges into small values because the reference is the EMS an average structure over the last 50 ns. The RMSD for DI and DIII converge quickly, and then evolve collectively. Note that only in the IS150 system the RMSD for DII presents low fluctuation

The analysis of the RMSD evolution of the three separated domains, with respect to EMS, suggests that only for the IS150 system the structure of all three domains evolve in concert toward the mean structure, while for the other cases, DII seems to be structurally relatively free from DI and DIII; Fig. 5. The domains DI and DIII move in phase relative to each other: they are adjacent and their globular conformations are, in general, stabilized by several β strands and salt bridges -even given that DI is fairly flexible. The RMSD evolution for system IS225 bears some similarity with that of IS150, however with higher fluctuations of DII and relatively smaller fluctuations of DI and DIII, Fig. 5.

**Fig. 6:**
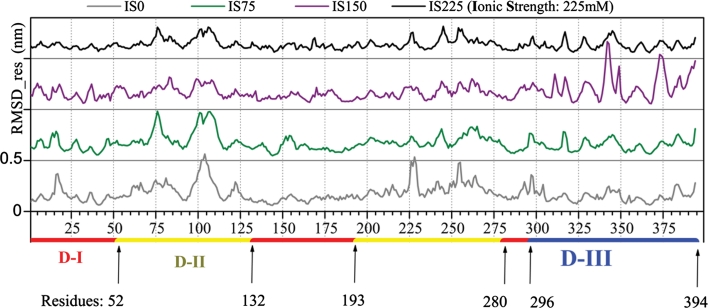
RMSD per residue for the four systems (EMS was used as reference). The sequence of residues belonging to domains DI (*red*: residues 1–52; 133–193; 281–296), DII (*yellow*: residues 53–132; 194–280) and DIII (*blue*: residues 297–394) are identified at the *bottom* of the figure. Changes on the ionic strength perturb structurally the location of several residues. DI and DII nearly preserve their overall fluctuation pattern, presenting systematically smaller local configurational perturbation as the ionic strength changes from 0 to 225 mM (RMSD scale runs from 0 up to 0.5 nm for each case). However, under the system IS150 conditions (*purple line*), the residues from 340 to 350, and the region around residue 375, all belonging to DIII, are significantly perturbed

Internal individualized conformational behavior, as well identification of regions of greater or lesser configurational changes can be seen through the RMSD per residue (RMSD_res) relative to EMS, plotted in Fig. 6 for all four systems. In all cases, the residues of DI fluctuate in an almost uncorrelated fashion and with small amplitude, confirming its intrinsic conformational stability; Fig. 4b. For systems IS0 and IS75, it is observed that in the fusion peptide region, particularly around Gly100 (cd loop), a sequence of residues fluctuate together, whose RMSD_res reaches values about 0.5 nm; in the other two systems, IS150 and IS225, it stands at about 0.3 nm. The residue sequences from Ala224 up to Asn230, and from Val250 up to Ile270 are regions of DII that, especially under the conditions of the IS0 system, are unstable with respect to the EMS configuration. On the other hand, under the conditions of the IS150 system, the DIII, although presenting highly correlated moves, like a rigid body (Fig. 4b), becomes particularly soft around some residues with RMSD_res reaching the largest values, mainly for those residues that are in contact with the solvent. Note that 150 mM is the ionic strength of the extra cellular medium, where the E protein firstly operates. Therefore, preservation of the basic structure topology, correlated internal moves, flexibility and extensive structural change are characteristics of the E protein at acid pH, which can be delicately modulated by the ionic strength. Particularly, such properties seems to be relevant for DIII, for example, which must present some degree of flexibility to interact efficiently with its receptors, and is the domain that contains sites that discriminate among different types of DENV, as well as sites that form the complex antibody-reactive protein [10, 21, 22].

**Fig. 7:**
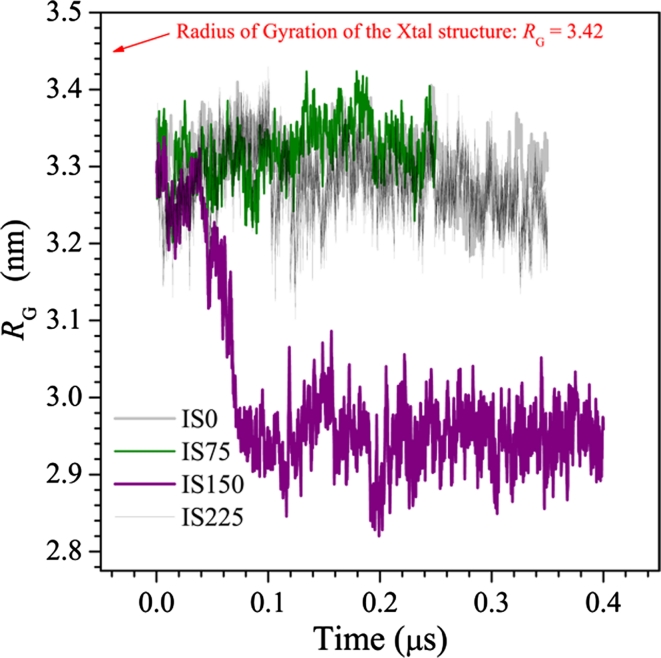
Evolution of the radius of gyration *R*_G_ of the E protein. Only under the conditions of the system IS150 the *R*_G_ is significantly reduced by about 10 % (reducing about 30 % of the protein volume)

The E protein structure is more compact at 150 mM than at smaller or larger ionic strengths, as shown by the evolution of its radius of gyration R_G_ in Fig. 7 (see also Fig. 3a). Consistently, at this condition it presents the smallest distal length *D*_max_ and the smallest mean radius of gyration *R*_G_, Table 3 (see also Table 2). The *R*_G_ remains stable around 3.30 nm for systems IS0, IS75 and IS225, but in the IS150 system it is reduced to 2.96 nm, contrasting with the most extended experimental structure (R_G_ = 3.42 nm). This increased compaction must be considered as the main difference between the IS150 structure and the others, because it reveals the degree of flexibility of the E protein, which can be important in the processes that conduct to the docking of the E protein on the cellular membrane [15]. The above results reveal that some critical points of the E protein are liable to make extensive moves. This is in agreement with the idea that the flexibility of E protein is a functional requirement for assembly and infection of flaviviruses[22]. For example, considering the E protein laying flat on the viral surface, the reduction of the angle ϕ raises the end of DII, exposing the fusion peptide to the medium.

**Table 3:** Average distal length *D*_max_ and radius of gyration *R*_G_ for all systems studied

	Xtal	ISO	IS75	IS150	IS225
A					
*D*_max_ (nm)	12.0	11.5	11.9	**10.1**	11.7
B					
*R*_*G*_ (nm)	3.42	3.31	3.32	**2.96**	3.29

The Xtal structure is the longest one: *D*_max_ = 12.0 nm and *R*_G_ = 3.42 nm. *D*_max_ and *R*_G_ reach the smallest values for the IS150 system (bold figures)

Furthermore, our findings suggest that such flexibility is modulated by environmental thermodynamic intensive parameters. However, in order to complete these results, MD simulations were performed for basic pH (with all histidine residues in the unprotonated form). The simulations were performed for 0.4 μs, for all four systems, that is, IS0, IS75, IS150: In all cases, the E protein structure was very stable, basically preserving its original crystallographic structure. Therefore, these two complementary results together, at acid and basic pH, constitute a strong support to the histidine switch hypothesis [23, 24], that is: histidines, being the only amino acids that had their protonation state changed, constitute the main factor that activates the expressive conformation change of E Protein.

### Energy analysis

#### The hydrogen bond networks

Hydrogen Bonds (HB) in E protein were identified by energetic criteria [28, 29, 40]; about 50 % of them are involved in the formation of secondary structures: 182 HB are found in the Xtal structure, as well as in the IS0 and IS75 systems structures. In the other two systems (IS150 and IS225) there are 187 HB. Table 4 shows that the ensembles of residues that constitute the secondary structures in all four systems are very similar to that found in the crystallographic structure. However, it must be noted that not all the sheets and helices are fully rediscovered in the structure of the monomer in aqueous solutions at acid pH. The largest differences are observed in IS225: it presents loss of structure in the β3, and β8 sheets (compare Tables 1 and 4), but exhibits a new sort of α helix formed by the residues Arg210–Phe212. While in the IS150 system the Asn103 residue forms only one HB with the residue Arg73, in all the other systems a shift in the positions of the HB is observed since the HBs of the antiparallel β sheet are found between Pro60–Arg73 with Gly112–Asn124. In IS150, this structure exists only in the Pro60–Leu65 with Phe119–Asn124 region. A similar shorter structure has already been found [9]. Also in the IS150 system, a new β antiparallel sheet is created in the Gly100–Phe108 region.

**Table 4:** The constitution of the secondary structures for crystallographic and all four systems structures

Type	Xtal		ISO		IS75		IS150		IS225	
β+	9–13	30–34	9–13	30–34	9–13	30–34	8–13	29–34	8–13	29–34
β−	20–25	283–288	20–25	283–288	20–25	283–288	20–25	283–288	20–25	283–288
β−	31–33	41–43	31–33	41–43	31–33	41–43	–	–	–	–
β−	41–45	139–143	41–45	139–143	41–45	139–143	40–45	139–144	40–45	139–144
β−	47–50	135–138	47–50	135–138	47–50	135–138	47–50	135–138	47–50	135–138
β−	54–72	113–129	–	–	–	–	–	–	–	–
β−	57–59	220–222	57–59	220–222	57–59	220–222	57–59	220–222	57–59	220–222
β−	62–66	118–123	62–66	118–123	62–66	118–123	62–65	119–123	62–65	119–123
β−	69–72	113–116	69–72	113–116	69–72	113–116	–	–	–	–
β−	90–99	109–118	90–99	109–118	90–99	109–118	90–97	111–118	90–97	111–118
β−	–	–	–	–	–	–	99–101	106–108	99–101	106–108
β−	126–128	198–200	126–128	198–200	126–128	198–200	126–127	199–200	126–127	199–200
β−	138–141	160–163	138–141	160–163	138–141	160–163	138–141	160–163	138–141	160–163
β−	171–175	179–183	171–175	179–183	171–175	179–183	171–175	179–183	171–175	179–183
β−	182–186	284–288	182–186	284–288	182–186	284–288	182–185	285–288	182–185	285–288
β−	196–200	205–209	196–200	205–209	196–200	205–209	196–200	205–209	196–200	205–209
β−	205–207	268–271	205–207	268–271	205–207	268–271	205–207	268–271	205–207	268–271
αH	–		–		–		210–212		210–212	
β−	238–241	249–252	238–241	249–252	238–241	249–252	238–241	249–252	238–241	249–252 |
αH	256–260		256–260		256–260		255–259		255–259	
β−	306–308	324–326	306–308	324–326	306–308	324–326	305–308	324–327	305–308	324–327
β−	320–324	365–369	320–324	365–369	320–324	365–369	320–324	365–369	320–324	365–369
β−	337–339	378–380	337–339	378–380	337–339	378–380	337–339	378–380	337–339	378–380
β−	350–351	369–370	350–351	369–370	350–351	369–370	–	–	–	–
β−	374–380	387–393	374–380	387–393	374–380	387–393	375–380	387–392	375–380	387–392

There are one parallel (β+), 22 antiparallel (β−) sheets, and two small α-helixes, one of which exists only for specific ionic strengths, namely IS150 and IS225

#### The protein internal energy

The ionic concentration used in experimental conditions has a direct consequence on the Coulomb interactions between residues and their surroundings. In the qualitative interpretation of the scope and intensity of Coulomb interactions, the Debye-Hückel model underlines that the interactions between net electrostatic charges are almost completely shielded at 0.64 nm for the ionic strength of the IS225 system; 0.78 nm for IS150; 1.11 nm for IS75, and a practically infinite distance for the IS0. Although purely Coulomb interaction energies are very sensitive to the concentration of the ions, differences in ionic strengths did not result in superficial charge distributions obviously different for a protein in solution. The intra-molecular interaction energy is about 7 % more attractive in the IS0 (−3,180 kcal/mol) than in the other three systems (around −2,975 kcal/mol).

At the beginning of the fusion peptide (DII), a region formed by the highly charged or polar residues, namely Asn83, Glu84, Glu85, Gln86, Asp87, Lys88 and Arg89, *E*_intra_ is very attractive in IS0, IS75 and IS150, but less attractive in IS225. At the other extreme of the fusion peptide–the hydrophobic region Gly111–Thr120, the intra-molecular interactions become repulsive, as well as along its Asp215–Gly223 sequence. It is interesting to note that the residue sequences Asn83–Arg89 and Gly111–Thr120 are exposed to the solvent, while the stretched sequence Asp215–Gly223 is internal. The consequence is a particular stability of the region of the fusion peptide, necessary for this fragment to be in a favorable position to undergo chemical reactions with target groups. The stability of the peptide fusion seen through the protein’s internal energy does not imply a lack of overall flexibility detected by RMSD_res, Fig. 6, which must be a collective motion of particular regions between *relatively fixed sites.*

The tertiary structure of the E protein is energetically better described through the mean interaction energy, as shown in Table 5, which lists the average intra- and inter-domain, total potential energy, and the corresponding average potential energy per pair of interacting residues. The inter-domain energy depends mainly on the distance between domains, while the total intra-domain interaction energy depends directly on the number of residues in the domain, which are 129, 167 and 98 residues in DI, DII and DIII, respectively. Intra and inter-domain average energies are fairly constant with respect to the ionic strength, as well as the mean interaction energies of the residues. However, they present a subtle oscillatory behavior in function of the ionic strength, with the energies for the IS150 system being located, in general, at a local maximum. Inter-domain energies are expressively weaker than intra-domain energies; particularly, the DII/DIII interaction is practically nil due to the distance between them.

**Table 5:** The mean interaction energies (kcal/mol) between pairs of domains and in each domain

	ISO	IS75	IS150	IS225
D-I	−4,407(8)/−0.53	−4,541(9)/−0.55	−4,504(8)/−0.55	−4,476(8)/−0.54
D-I/D-II	−227(5)/−0.01	−241(4)/−0.01	−200(4)/−0.01	−208(5)/−0.01
D-I/D-III	−274(6)/−0.02	−277(5)/−0.02	−243(5)/−0.02	−234(6)/−0.02
D-II	−5,648(8)/−0.53	−5,690(8)/−0.53	−5,656(8)/−0.53	−5,678(8)/−0.53
D-II/D-III	−21(7)/0.00	0/0	0/0	0/0
D-III	−3,325(8)/−0.70	−3,358(8)/−0.71	−3,294(8)/−0.69	−3,333(8)/−0.70
Total energy	−13,902	−14,107	−13,897	−13,929

The respective standard deviations are shown in parenthesis; and after the slash are shown the mean interaction energy by pair of residue. Note that although being the smallest domain, DIII presents the largest average inter-domain energy (over 30 % more than DI and DII), in agreement with its observed conformational stability

An important aspect of the energy of the E protein, depicted in Table 5 is that at acid pH, and when the system state is closer to physiological conditions (IS150 system), one gets always one less cohesive molecule than in the other system conditions, as well as for almost all inter- and intra-domain energies. Such an observation should be directly related to the activity of this protein that is favored by a somewhat lower stability.

#### Interaction residue-ions

**Fig. 8:**
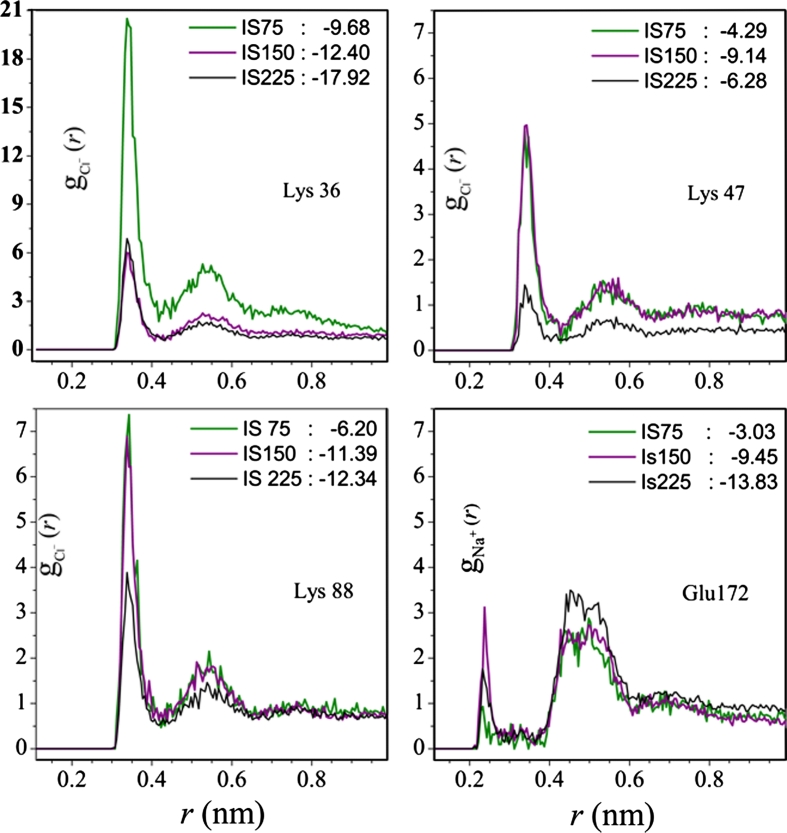
Radial distribution functions of the counter-ions of Lys36, Lys47, Lys88, Glu172. In the *insets* are shown the corresponding interaction energies (kcal/mol)

The influence of the ionic strength on the residue-ions interactions energies, *E*_ion_, is to be searched through the interactions of the ions with, mainly, the lateral charged groups of the thirteen Arg, sixteen Asp, thirty one Glu, eleven His and thirty three Lys residues found in the E protein. The residues Lys64, Lys123, Lys202 and Lys246 and Lys247 are those that show the strongest interaction with the ions in all four studied systems. These residues are located on the surface of DII, exposed to the solvent, just on the opposite side of the fusion peptide. Lys64 pertains to β9 (see Table 4) while the other cited Lys residues are not included in any secondary structure, so that they are fairly free to move. The strongest residue-ion interactions are detected in DII: *E*_ion_ = −14.5; −20.1 and −25.6 kcal/mol for IS75, IS150 and IS225 conditions, respectively. The three strongest residue-ions interactions in the two other domains are: *E*_ion_ = −8.9; −12.6; −14.7, and −5.2; −9.2; −14.7 kcal/mol, for DI and DIII respectively. In such interactions with ions, the more energetically important residues are, in order: Lys, Arg, Glu, Asp and His. His244 is of particular interest, as is the unique His that presents *E*_ion_ = −7.64, −8.29 and −11.67 kcal/mol, in the IS75, IS150 and IS225 systems, respectively. The effect of the ionic strength in the residue-ions interactions is better understood through a careful analysis of the repulsion/attraction effect of counter- and co-ions all together with the structural extensive changes. This can be seen by the oscillating pattern of *E*_ion_ in function of the ionic strength: the residue-ions interactions in IS75 system, *E*_ion_(IS75), for DI, DII and DIII, is greater (that is, less cohesive) than such interaction in IS150 and in IS0: *E*_ion_(IS75) > *E*_ion_(IS0), as well >*E*_ion_(IS150). On the other hand, we found that for DII and DIII, *E*_ion_(IS150) < *E*_ion_(IS225) (more cohesive), but for DI, *E*_ion_(IS150) > *E*_ion_(IS225) (less cohesive). Such behavior can be ascribed to local fluctuations, but the case of Lys47 is amazing: Lys47 is located in the internal portion of the hinge between the DI/DIII and DII, and *E*_ion_ are −3.66(IS75), −7.43(IS150) and +0.17 kcal/mol (IS225). Two other residues [Lys160 and Lys204 with *E*_ion_ = −2.11(IS75), −5.24(IS150) and +1.64(IS225), and *E*_ion_ = −7.99(IS75), −11.24(IS150) and −6.45 kcal/mol (IS225)] are located at 1.4 nm from Lys47, so that they are submitted to the direct interactions of the same ions. Another residue, Lys157, is at 1.6 nm from Lys47 and 0.7 nm from Lys160; and *E*_ion_ = −7.4(IS75), −12.6(IS150) and −4.1(IS225) kcal/mol. Consequently, the internal structure of the hinge located between the DII and DI/DIII allows for detecting conformational fluctuations that are of primordial importance for the necessary conformation changes during the trimer formation and the following interactions with the cellular membrane. However, such behavior is a consequence (rather than a cause) of conformation changes of the hinge region, when the ionic strength varies from 150 to 225 mM. The decrease of exposure of the Lys47, Lys157, Lys160 and Lys204 to the external solution reduces the residue interaction energies, making their neighborhood more cohesive, while their interaction with the solvent becomes weaker. Therefore, the hinge between the DI/DIII and DII is less stable in IS225 than in IS150. Radial distribution functions, *g*_ion_(*r*) for Lys36, Lys47, Lys88 and Glu172 are shown in Fig. 8; *r* is the distance between the geometric center of the charged group and the center of the corresponding ions. The influence of the neighborhood of the residues on the ion distribution can be clearly seen in both: the profiles *g*_ion_(*r*) itself, and through the corresponding interaction energies.

The geometry and size of charged groups, namely ammonium, guanidine groups and imidazolium moiety in Lys, Arg and His, respectively, result in $g_{{{\text{Cl}}^{ - } }} (r)$ that are similar for the same residues, but different for unequal ones. ${g_{\text{Cl}}} {(r)}$ for Lys presents two peaks with maxima at 0.34 and 0.55 nm (Fig. 8), but for His they are found at 0.41 and 0.62 nm, and $g_{{{\text{Cl}}^{ - } }} (r)$ for Arg presents peaks at *r* = 0.40 and 0.60 nm. The different heights of the peaks of $g_{{{\text{Cl}}^{ - } }} (r)$ correspond to different numbers of first and second chlorine neighbors of the charged groups. The first peaks, despite their good resolution, are due to a maximum of 0.08 ions (an exception is Lys64 in IS225 that presents 0.1 ions in this first peak). The numbers of chloride ions in the first two layers are listed in Table 6 for the His and Arg residues, and in Table 7 for Lys. We selected the layers that are at least 10 % occupied by ions.

**Table 6:**
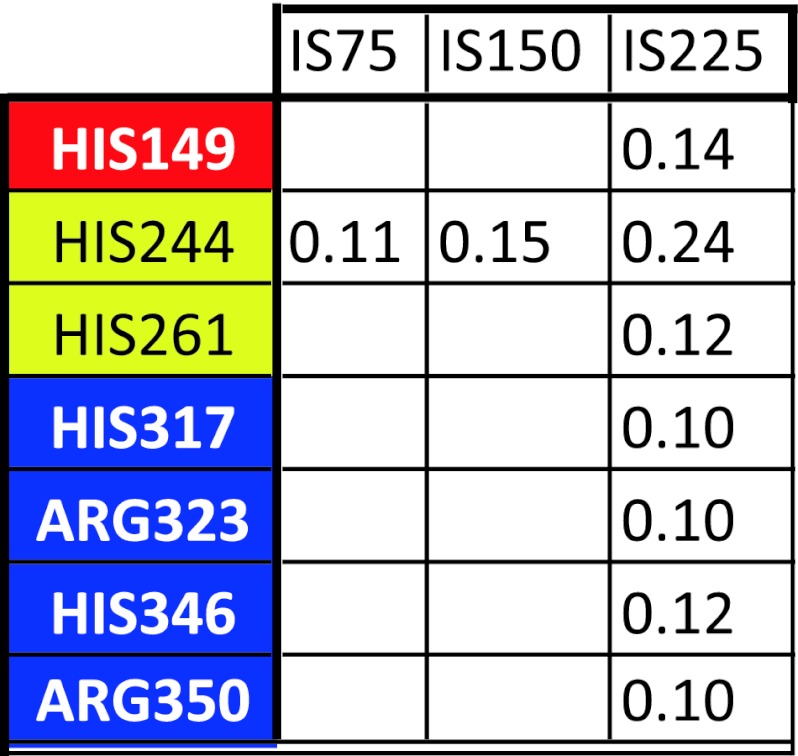
The mean numbers of chlorine ions in the first and second shells of the charged groups of the His and Arg residues that are at least 0.1

The residue domain is identified by the conventional colors (DI, red; DII, yellow; and DIII, blue). Only in DII the two first shells are significantly populated for IS75 and IS150 systems

**Table 7:**
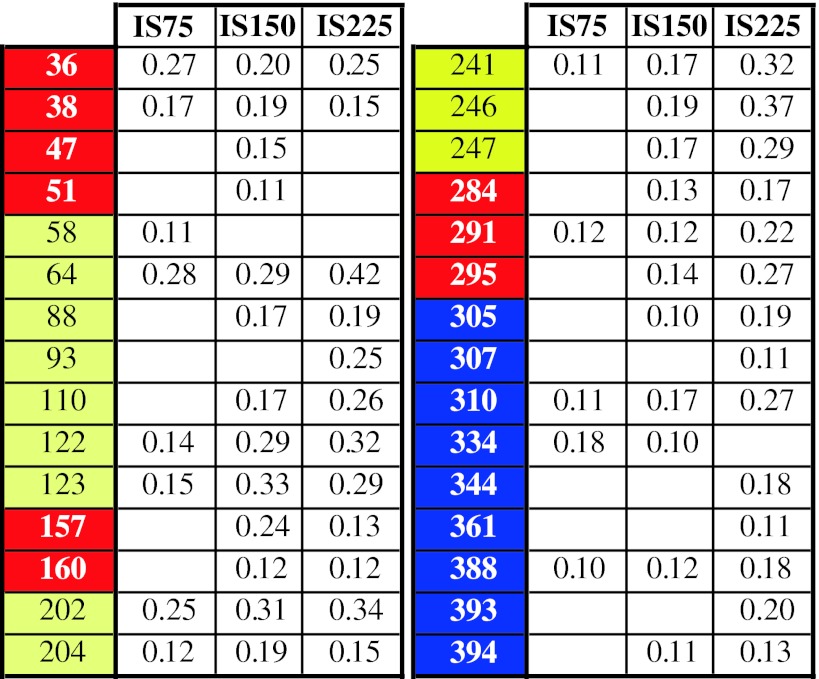
The mean numbers of chorine ions in the first and second shells of the charged Lys residues that are at least 0.1

The domain they pertain are conventionally colored as: DI, red; DII, yellow; and DIII, blue. Only in the IS225 system the relevant presence of chlorine ions in the two first shells are less affected by the locus of the residues

The butyl ammonium chain in the Lys residues is flexible and can accommodate easily into aqueous medium, being, therefore, more accessible to ions than the large and less flexible groups, such as guanidine and imidazolium groups of Arg and His. The influence of the increased number of ions on the different systems is made evident by looking at the His and Arg residues: since an ionic layer was identified only in the IS225 system with the exception of His, which is well exposed to the external field at the end of the DII. The Lys residues that have an ion shell are distributed more or less equally across all three domains, but the Lys residues in the 53–132 regions of DII have mostly the largest ion shell occupancy. This region is on the outer surface exposed to the aqueous environment and contains the fusion peptide. The consequence is that the influence of ionic strength on DII is the largest possible.

The negatively charged residues, Glu and Asp, contain the same COO^−^ group. Therefore, the $g_{{{\text{Na}}^{ + } }} (r)$ distributions present peaks at the same positions about 0.22 and 0.48 nm. In some cases, peaks are observed in both positions or only in the second, such as for Asp362 of the IS75 system, as shown in Table 8, which lists the mean number of ions in the first two ionic shells. Note that IS75 shows no case of an ionic shell containing on average at least 0.1 Na^+^ ion, while some residues of the IS150 and IS225 systems present a significant number of ions in such a layer. The two residues, Asp and Glu, are those with the most attractive solvation energy [41]: this explains why these residues do not hold well defined ionic layers, which suggests that the cations from the surrounding environment have a minor role.

**Table 8:**
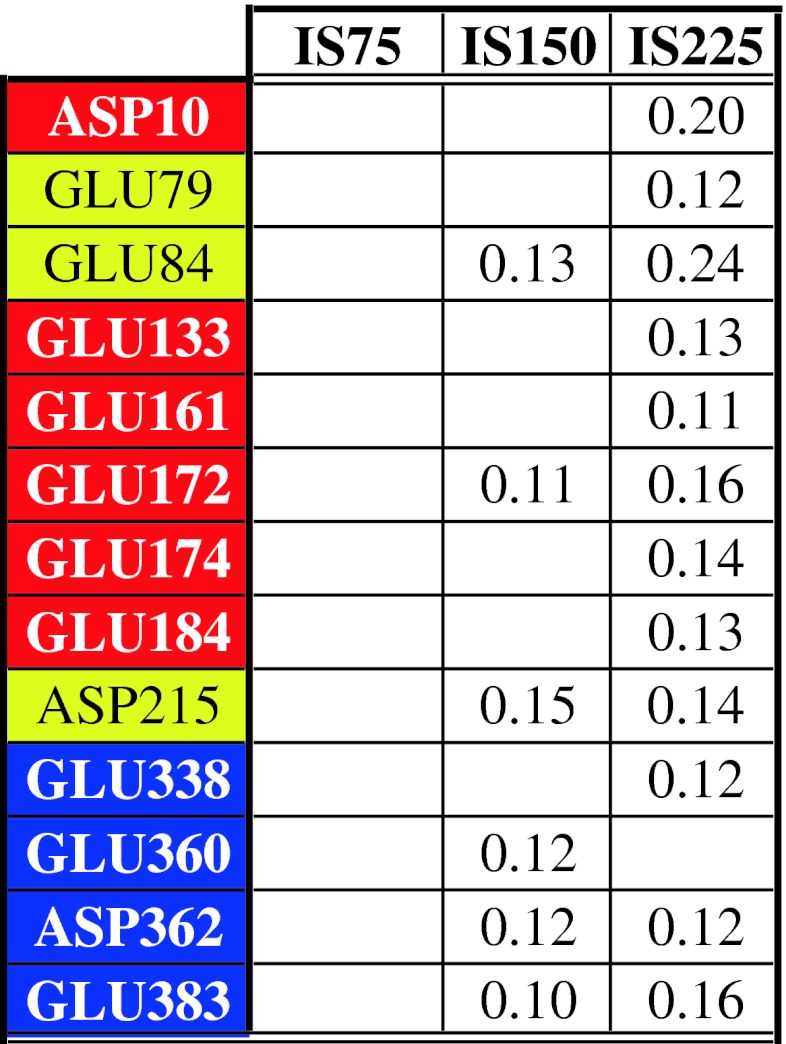
The mean numbers of sodium ions in the first and second shell of the charged groups of the Asp and Glu residues that are at least 0.1

Conventional colors identify the domains. Note that in the system IS75 no shells are significantly populated

#### The protein–solvent interaction

The numbers of HB between atoms of the protein and the solvent, ${\text{HB}}_{{{\text{H}}_{ 2} {\text{O}}}}$, using the criterion of occurrence larger than 0.2, are 138, 139, 136 and 126 in IS0, IS75, IS150 and IS225 systems, respectively. All these ${\text{HB}}_{{{\text{H}}_{ 2} {\text{O}}}}$ are promoted by solvation of charged groups, namely residues Arg, Asp, Glu, His, and Lys. The mean occurrence values are very similar for the four ionic strength systems (about 0.33), so that the ionic strength of the medium does not influence the solvation of the charged and uncharged residues. The weakness of the cation layers can now be explained since the average energy of interaction between the oxygen atoms of the COO^−^ groups and water molecules of solvation is too attractive (around −30 kcal/mol) to be disturbed by interactions with the ions. Therefore, the screening of the electrostatic interactions, increased with the system ionic strength, has a slight influence on the potential energy of the protein, and the main contribution to the total energy of the protein is the interaction with the solvent.

**Fig. 9:**
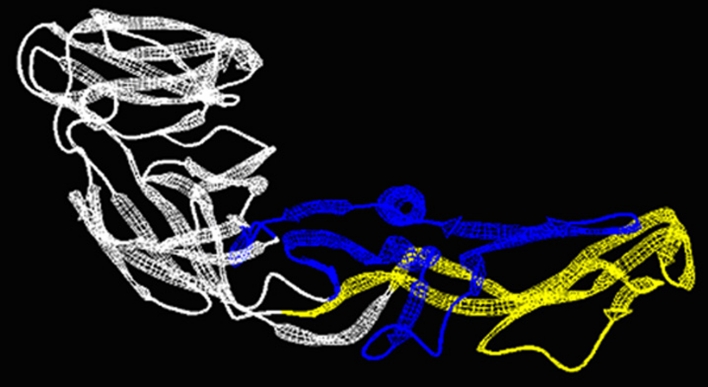
The DII decorated from Cys60 to Gln130 in *yellow*, and from Asn194 to Thr280 in *blue* (IS150 system)

It should be noted that the amphipathy of the fusion peptide segment is low: from Phe90 up to Cys121, the amphipathy remains mainly in the range 0–21—with a peak at 69 for Lys110, being the mean amphipathy [41] equal to 12. This means that the interaction energy between the side groups and the solvent is −13.9 kcal/mol, on average. This energy provides, nevertheless, the insertion of this segment in the solvent. The region Cys60 up to Gln130 is shown in Fig. 9 highlighting its exposure to the solvent, which remains in the dimer configuration [22–26].

Each of the interactional details analysed here has local consequences that are not hard to understand; but, the global effect of all such interactions, which interfere one another, is much more difficult to describe but can be systematically done by MD simulation. Indeed, looking for specific residue interactions and residue-solvent interaction, responsible for (in)stability and pH sensing, for example, new insights can be gained [42] and recursively tested.

## Final comments and conclusions

PH and ionic strength modulate the structural conformation of the E protein of DENV. A set of far-reaching runs MD simulations of the monomer of E protein in solution at 300 K, carried out at four distinct ionic strength levels, pointed out that, exclusively at ionic strength of 125 mM (the closest to the physiological condition) and acid pH (all histidine fully protonated), the protein undergoes a significant structural variation: the angle ϕ, determined by the relative orientation of the domains DI/DIII and DII, is substantially reduced (about 25°). This transition is emphatically shown by the RMSD evolution with respect to the experimental structure and the ensemble mean structure (EMS), and changes on the Radius of Gyration. After such extensive structural change, occurring at time about 0.05 μs, the E protein conformation remains virtually unchanged until the simulation end, at 0.4 μs. These results suggest that the E protein can make extensive moves, modulated by environmental thermodynamic intensive parameters.

The above results were confronted with the simulation results for basic pH, with the ionic strength varying from zero to 225 mM. In all these extra runs the protein conformation remained pretty stable, maintaining its original crystallographic structure substantially unperturbed. Therefore, we have that at basic pH, when all histidines are umprotonated, the E protein monomer preserves its conformation, just like it appears in the dimer arrangement, while at acid pH, with all histidines protonated, the monomer undergoes a extensive conformational change. This configuration change, subtly unleashed by reducing pH, supports the histidine switch hypothesis, which has been anticipated in order to explain the large conformation change the E protein suffers during the infection process [23, 24]. Indeed, the histidines are the only amino acids that undergo protonation precisely in the pH range between 6 and 7, constituting the exclusive factor in our simulation set that induces the expressive change on the conformation of the E Protein.

A complementary principal component analysis points out that, for the system IS150 under acid pH, the DIII is the most topologically rigid domain of E protein. On the other hand, DII is malleable in its central region, but its extremes—where on one side the fusion peptide is located and in the other is the hinge connecting DI/DIII and DII—are much less flexible. In its turn, the DI is fairly flexible in almost its full extent, although its basic structural topology is recursively maintained during all time (see Figs. 4b, 6). The domains and inter-domains relative malleability has been considered as a substantial property of E protein during the preliminary processes of membrane fusion, induced by acidification of the medium [25, 26]. However, according to our present results, no such conformational transition is observed (or at least becomes much less probable) at a lower (IS0 and IS75) or higher (IS225) ionic strength. Instead, in such conditions different than that of the IS150 system, the extended DII, deeply in contact with the environment, presents an unstable RMSD (Fig. 5), suggesting that, under such conditions, it is relatively freer to explore the local accessible configurational space. The effects of stretching caused by electrostatic interactions are the limiting factors for further compression of the monomeric E protein in the IS225 system. In its turn, the decreases of the angles ϕ cannot be due to the direct interactions between the DI/DIII and DII because, initially, they are too far apart and these interactions are too weak to result in large changes in the protein structures. Therefore, with the E protein becoming more flexible through the hinge, the DII could approach DI/DIII by chance, and then a new set of interactions take place, stabilizing the molecule in a more compact conformation.

The analysis of the intra-molecular HB shows that a quite complex set of interactions, involving about 180 HB in the formation of secondary structures, ensure a peculiar conformational stability for each domain of E protein, which preserve the basis of the topology of the three domains of E protein. Looking specifically at the domains, it is seen that they are maintained very efficiently by attractive interactions, including some salt bridges with strong cohesive energy of about −5 kcal/mol, or less. Interactions of the protein with ions of the solution are centered on the charged groups and on the ionic atmospheres in their neighborhood that are never very stable, indicating that the competition with the solvent for the attractive forces centered on the charged groups are in favor of the solvent. But, the aggregator or disruptive effect of the ions is among those responsible for the observed changes on the original structure of the protein, seen that they depend on ionic strength. The profiles of the protein/solvent interaction energy are not yet very clear, but the fusion peptide region always has a different behavior since this region has much less attraction for the solvent than the rest of the molecule. Finally, the calculated total potential energy suggests that the protein would be less stable in the IS150 system. This result indicates that the protein in the IS150 system can more easily undergo changes in its structure to better adapt to environmental conditions during cell attachment, or in the formation of the trimer. As it became evident, under the IS150 system conditions, DII and DIII present different behaviors: DII becomes stiffer and DIII present large fluctuation at specific residues, in comparison with the other tree systems (Fig. 6). Since these two domains have their importance in the specific case of membrane fusion (DII), and in the differentiation among virus serotypes and interaction with antibodies (DIII), alternative possibilities should be considered with regard to the use of this information: Even with the tactical malleability of DIII, but considering that the disease may develop with the variant of this domain, one may think that focusing on the search for antiviral substances in the region of DIII could not be the best solution. Instead, D-II presents a very elongated surface, presenting a region of residues full conserved among all serotypes, as well as a flexible region appropriated to be subjected to tests changing of stability and activity, through bonding to appropriate molecules.

In this work, the conformational behavior of the E protein monomer of the DENV-2 was focused mainly because of its multifunctional characteristics. The set of all simulation presented here—summing more then 2.5 μs, and the extensive conformational/energy analyses, although not constituting a definitive result, suggests that the conformation behavior of the monomer of E protein in acid solution is delicate, since that it depends on a definite ionic strength of the solution. Therefore, even though a great deal of work must yet be completed to have a full view of its role in the virus-host interaction, insights and alternative ideas are already possible. From the mechanism point of view, for instance, one could ask and try to answer, by simulation, whether the trimer formation could not be started by a rearrangement of disassembled dimers on the virus surface, as suggested by the cryoelectronmicroscopy and crystallographic structures of the trimer [5, 7], or if the membrane is really a necessary catalyzer for efficient formation of trimers [43].

Finally, *we hope that our findings will shed some light* on the molecular behavior of the E protein, thus contributing to the search for antiviral substances, interfering in the process of attachment of the virus in the cell, or effectively disrupting the process of the membrane fusion.
